# Cisplatin resistance in ovarian cancer: exploration and insights from metal ion homeostasis imbalance

**DOI:** 10.3389/fcell.2026.1852307

**Published:** 2026-06-09

**Authors:** Chong Chen, Yaqing Li, Yu Zhang, Sailong Liu, Yang Li, Luyan Shen

**Affiliations:** 1 First Hospital of Jilin University, Jilin University, Changchun, China; 2 Key Laboratory of Pathobiology, Department of Pathophysiology, Ministry of Education, College of Basic Medical Sciences, Jilin University, Changchun, China; 3 Experimental Teaching Center of Basic Medicine, College of Basic Medical Sciences, Jilin University, Changchun, China; 4 Department of Physiology, College of Basic Medical Sciences, Jilin University, Changchun, China

**Keywords:** cisplatin resistance, metabolic reprogramming, metal ion homeostasis, ovarian cancer, redox

## Abstract

Ovarian cancer remains a lethal gynecological malignancy, and cisplatin resistance is a major therapeutic barrier. Beyond classical mechanisms, dysregulated redox homeostasis is now recognized as a key determinant of cellular fate and chemoresistance. Metabolic reprogramming in cancer cells critically perturbs the cellular redox system, positioning mitochondria—a nexus of metabolism and redox signaling—as a promising therapeutic target for modulating platinum sensitivity. Importantly, metabolic reprogramming involves alterations in mitochondrial homeostasis of the redox-active copper (Cu) and iron (Fe) ions. These ions are fundamental to energy metabolism, and their dysregulation profoundly affects cellular stress responses. Critically, disrupted Cu/Fe homeostasis directly regulates novel cell death pathways: excess Cu induces cuproptosis through the aggregation of lipoylated tricarboxylic acid cycle proteins, whereas aberrant Fe metabolism drives ferroptosis through iron-dependent lipid peroxidation. Therefore, this review focuses on mitochondrial redox regulation and explores the connections among metabolic reprogramming, mitochondrial ion metabolism, and novel modes of programmed cell death in cisplatin-resistant ovarian cancer cells. From an ion-centric perspective, it aims to provide new insights into targeting the mitochondria-mediated redox regulatory network to enhance sensitivity to platinum-based drugs.

## 1 Introduction

**FIGURE 1:**
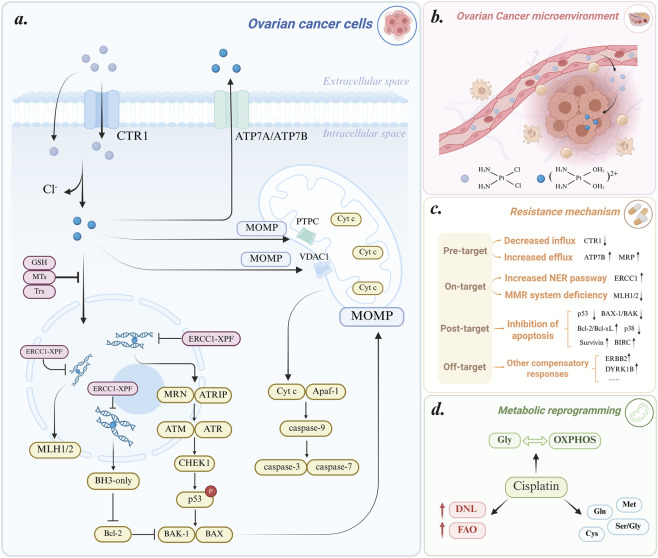
Cisplatin action mechanism, drug resistance and association with metabolic reprogramming. **(a)** Cisplatin enters ovarian cancer cells primarily through CTR1. In the intracellular low-chloride environment, it undergoes aquation to form the hydrated species [Pt(NH_3_)_2_(H_2_O)_2_]^2+^, which enters the nucleus. The active platinum species then covalently binds to the N7 position of guanine residues on DNA, predominantly forming 1,2-intrastrand or interstrand cross-links. This leads to severe distortion and conformational changes in the DNA double helix. The DNA damage is first sensed by ATM/ATR proteins, which are rapidly activated and trigger apoptotic signaling pathways mediated by CHEK1, p53, and other factors. This induces MOMP, promoting cytochrome c release and subsequent activation of the caspase cascade, ultimately leading to programmed cell death. Molecules containing thiol (–SH) groups, such as GSH, MTs, and Trx, can tightly bind to platinum atoms, inactivating them and preventing further DNA interaction. Following DNA cross-link damage, the cell first activates the NER pathway. The ERCC1-XPF nuclease complex—a key executor of NER—is recruited to the damage site, where its XPF subunit performs specific incision of the damaged DNA strand to initiate repair. The MMR system recognizes cisplatin-induced DNA lesions and can initiate apoptotic signaling. If the MMR system is deficient (e.g., due to downregulation of MLH1 or MSH2), cells may fail to detect DNA damage and activate the suicide program. Additionally, ATP7A and ATP7B contribute to cisplatin efflux, reducing intracellular drug accumulation. **(b)** Cisplatin exists in different chemical forms in the ovarian microenvironment and acts on ovarian cancer cells. **(c)** Based on functional and mechanistic levels, cisplatin resistance can be categorized into four tiers: Pre-target level: Downregulation of CTR1 reduces cisplatin influx. Target level: Upregulation of ERCC1 enhances NER. Post-target level: Dysregulation of apoptosis pathways (e.g., *via* p53 inactivation).Off-target level: Compensatory responses (e.g., mediated by ERBB2 signaling). **(d)** Cisplatin induces metabolic reprogramming in ovarian cancer cells, altering the balance between Gly and OXPHOS. For example, it may suppress mitochondrial function, promoting a shift toward aerobic glycolysis. Additionally, cisplatin is associated with increased *de novo* lipid synthesis and altered metabolism of amino acids (e.g., glutamine), which support cell survival under drug-induced stress. (Figure was created with BioRender.com). Abbreviations: CTR1, Copper transporter 1; ATP7A/ATP7B, Copper-transporting ATPases 7A/7B; MOMP, Mitochondrial Outer Membrane Permeabilization; PTPC, Permeability transition pore complex; VDAC1, Voltage-dependent anion channel 1; MTs, Metallothioneins; Trx, Thioredoxin; ERCC1-XPF, Excision repair cross-complementation group 1-xeroderma pigmentosum complementation group F; MLH1/2, Mismatch repair gene 1/2; ATRIP, ATR-interacting protein; ATM, Ataxia-telangiectasia mutated protein; ATR, Ataxia-telangiectasia and Rad3-related protein; CHEK1, Checkpoint kinase 1; NER, Nucleotide excision repair; MMR, Mismatch repair; MRP, Multidrug resistance-related protein; BIRC, Baculoviral IAP repeat-containing protein; ERBB2, Human epidermal growth factor receptor 2; DYRK1B, Dual-specificity tyrosine phosphorylation-regulated kinase 1B; DNL, De novo lipid synthesis; FAO, Fatty acid oxidation; OXPHOS, oxidative phosphorylation.

Ovarian cancer is the third most common gynecological malignancy worldwide and has the highest mortality rate among gynecological cancers (Kuroki and Guntupalli, 2020). Because of its deep pelvic location and nonspecific early symptoms, approximately 70% of patients are diagnosed at an advanced stage (III/IV), resulting in a very poor prognosis (Cheng et al., 2022). Although chemotherapy drugs such as cisplatin have shown some efficacy in ovarian cancer, cancer cells gradually develop a multifactorial, multistep drug-resistance network under drug selection pressure (Figure 1). It is currently understood that resistant cells respond to cisplatin stimulation through a series of adaptive homeostatic reactions, including enhanced drug efflux, upregulation of resistance-related proteins, and increased DNA damage repair capacity. Collectively, these responses interfere with the apoptotic signals, thereby establishing drug tolerance (Shen et al., 2018).

At the pre-target stage (Galluzzi et al., 2014), resistance arises from reduced drug uptake, such as downregulation of copper transporter 1/2 (CTR1/2), and increased efflux, such as overexpression of copper-transporting ATPases 7A/7B (ATP7A/7B), leading to decreased intracellular drug concentrations (Yoshida et al., 2013; Lukanović et al., 2020; Li et al., 2017). Concurrently, molecules such as glutathione (GSH) and metallothionein (MT) inactivate cisplatin by binding to it through their thiol groups (Criscuolo et al., 2022; Kawahara et al., 2019). At the on-target level, enhanced DNA damage repair is a key mechanism: high expression of factors such as excision repair cross-complementation group 1 (ERCC1) in nucleotide excision repair (NER) effectively repairs platinum-DNA adducts; mismatch repair (MMR) deficiency, for example, loss of mismatch repair gene 1/2 (MLH1/2), prevents cells from recognizing damage and initiating apoptosis; and restorative mutations in homologous recombination (HR) pathway-related genes, such as breast cancer associated gene 1/2 (BRCA1/2), can restore repair capacity and thereby affect cisplatin sensitivity (Zhang et al., 2021; Helleman et al., 2006; Press et al., 2008). Post-target resistance is mainly characterized by apoptotic escape: downregulation of p53 (Fraser et al., 2008), upregulation of anti-apoptotic proteins such as Bcl-2/Bcl-xL, and overexpression of inhibitor of apoptosis proteins (IAPs) collectively suppress cell death execution (Yuan et al., 2020; McCabe et al., 2014). Furthermore, off-target mechanisms of cisplatin resistance include sustained activation of epidermal growth factor receptor/erythroblastic leukemia viral oncogene homolog 2 (EGFR/ErbB2) signaling and dual-specificity tyrosine phosphorylation-regulated kinase 1B (DYRK1B) mediated antioxidant responses, both of which support cell survival under stress (Yang et al., 2020; Gao et al., 2012). During tumor cell killing, cisplatin-induced depletion of cellular reductants, cytochrome c release, and related consequences can trigger oxidative stress that gradually exceeds cellular tolerance, disrupts cellular structures, and accelerates cell death (Dasari and Tchounwou, 2014). Tumor cells, in turn, employ a range of adaptive mechanisms to counteract oxidative damage and maintain viability. Therefore, targeting the redox regulatory network of tumor cells has become a major research focus for reversing cisplatin resistance.

Metabolic activity serves as a central hallmark of tumor cell function and is intricately linked to proliferation, differentiation, survival, and dynamic crosstalk with the tumor microenvironment (Hanahan and Weinberg, 2011). Ovarian cancer cells, especially high-grade serous carcinoma cells (which account for approximately 70% of epithelial ovarian cancer cases), undergo profound alterations in multiple metabolic pathways in response to cisplatin exposure, using adaptive metabolic reprogramming to thrive in the hostile microenvironment and sustain robust proliferative capacity (Zhang et al., 2019; Zhang X. Y. et al., 2018) (Figure 1). As a core hub for cellular metabolism, mitochondrial function regulates the balance between glycolysis and oxidative phosphorylation (OXPHOS), coordinates key metabolic pathways such as lipid and amino acid metabolism, and maintains close crosstalk with diverse metabolic processes through mitochondrial redox homeostasis. Therefore, the role of mitochondria as a central hub in tumor cell metabolism and cisplatin sensitivity has attracted extensive attention. Studies have shown that activation of OXPHOS metabolism together with simultaneous upregulation of glycolysis is one of the mechanisms of cisplatin resistance (Pervushin et al., 2024). This metabolic plasticity enables ovarian cancer cells to flexibly adjust the glycolysis-OXPHOS balance in response to environmental changes. Under cisplatin selection, OXPHOS-dependent cell subpopulations are gradually enriched to form resistant clones characterized by decreased glucose uptake and *de novo* lipid synthesis, as well as increased uptake of exogenous fatty acids and mitochondrial fatty acid oxidation, thereby providing energy and survival advantages under chemotherapy-induced oxidative stress (Liu et al., 2025; Tan et al., 2022). It has been found that peritoneal metastatic ovarian cancer cells can take up fatty acids released by omental adipocytes and generate energy through β-oxidation (fatty acid oxidation, FAO) to support survival and proliferation under cisplatin stress (Nieman et al., 2011). Additionally, ovarian cancer cells exhibit relatively low expression of heat shock proteins (such as HSP70 and HSP90), which may compromise their stress-protection capacity under chemotherapy (Hoter and Naim, 2019); however, certain subpopulations compensate for this defect by enhancing glutamine metabolism and GSH synthesis, thereby resisting cisplatin-induced oxidative stress (Hudson et al., 2016). Notably, the execution of mitochondrial functions underlying these metabolic reprogramming processes is highly dependent on mitochondrial metal ion homeostasis. As cofactors and signaling molecules for various key enzymes, metal ions such as copper and iron affect dynamic redox balance by regulating mitochondrial metabolic enzymes and respiratory chain function. Therefore, given their critical regulatory role in mitochondrial oxidative stress, the effect of metal ion homeostasis on platinum drug action and cellular fate in ovarian cancer cells warrant substantial research attention.

The high heterogeneity of tumor cells limits the efficacy of reversing drug resistance through apoptosis-targeted strategies. In recent years, researchers have turned their attention to other forms of regulated cell death (RCD), such as cuproptosis, ferroptosis, and pyroptosis, exploring them as potential approaches to treat chemotherapy-resistant tumors. Ferroptosis is an iron-dependent form of RCD driven by lipid peroxidation, closely linked to glutathione metabolism and the lipid antioxidant system. Inhibiting glutathione peroxidase 4 (GPX4) or system Xc^−^ can induce this death pathway (Jiang X. et al., 2021). Cuproptosis, a newly identified RCD mode, is strongly associated with intracellular copper accumulation and mitochondrial respiratory chain dysfunction, with cells exhibiting active OXPHOS being particularly susceptible (Wang et al., 2022). These non-apoptotic RCD pathways intersect mechanistically with cisplatin resistance at the level of redox metabolism, energy metabolism, and related processes. Therefore, from the perspective of mitochondrial integration of material metabolism and ion homeostasis, they may offer new therapeutic opportunities to overcome cisplatin resistance in ovarian cancer.

This review systematically outlines cisplatin resistance in ovarian cancer through the lens of redox and energy metabolism. It then discusses how copper and iron metabolism can trigger cell death and help reverse drug resistance. Unlike previous reviews that focus separately on metabolic reprogramming, ferroptosis, or cuproptosis, this review integrates mitochondrial metal ion homeostasis with redox regulation. Our ion-centric perspective connects metabolic plasticity, mitochondrial dysfunction, and non-apoptotic cell death. We aim to reveal a previously overlooked vulnerability in drug-resistant ovarian cancer cells. Where ovarian cancer-specific evidence is missing, we refer to findings from other cancer models and clearly note their extrapolated nature.

## 2 The metabolism in cisplatin-resistant ovarian cancer cells and mitochondria

### 2.1 Metabolic reprogramming and ovarian cancer cisplatin resistance

Emerging evidences in tumor metabolism research have revealed that tumor metabolism is not as uniform and homogeneous as traditionally believed (such as the classic Warburg effect), but exhibits high heterogeneity and flexibility (Kim and DeBerardinis, 2019). Metabolic heterogeneity stems from the integrated regulation of internal factors such as gene mutations in cancer cells and external factors of the tumor microenvironment (TME) (such as hypoxia, nutrient deficiency, and intercellular interactions). Ovarian cancer cells with dynamically altered metabolic phenotypes participate in the formation and fine-tuning of the aforementioned classic chemoresistance mechanisms through the reprogramming of glucose, lipid, and amino acid metabolism, as well as the redox metabolic regulatory networks shaped by these adaptive changes. These cells further interact cooperatively with the TME to maintain survival under chemotherapeutic stress. This underscores the critical regulatory role of tumor cell metabolic flexibility in the mechanism of chemoresistance.

#### 2.1.1 Glucose metabolism

Most ovarian cancer cells are highly dependent on aerobic glycolysis (the canonical Warburg effect) to rapidly generate ATP and supply precursors for biosynthetic processes (such as glucose-6-phosphate), and are sensitive to glycolysis inhibition. Under hypoxic stress or oncogenic signaling, HIF-1α activation further upregulates the expression of multiple glycolytic genes such as GLUT1, LDHA, PFKFB3, and HK2 (Ma Y. et al., 2024; Nantasupha et al., 2021). This metabolic adaptation is critical not only for efficiently generating ATP to support for tumor growth but also for enhancing the pentose phosphate pathway (PPP)—which provides biosynthetic building blocks and generates copious NADPH. This sustains reduced GSH levels and partially attenuates cisplatin-induced oxidative stress. However, OXPHOS in mitochondria is not universally suppressed. Studies have shown that tumor stem cell (CSCs) subpopulations or cells selected by cisplatin may be more dependent on mitochondrial OXPHOS to produce substantial ATP (Sriramkumar et al., 2022; Emmings et al., 2019), supports efficient DNA repair (such as the NER pathway), drug efflux, and reactive oxygen species (ROS) scavenging (Furuta et al., 2002; Komatsu et al., 2000). Notably, both glucose metabolism and OXPHOS are precisely governed by intracellular metal ion homeostasis. For instance, iron ions are used to synthesize iron-sulfur clusters and heme, which participate in the assembly of aconitase in the tricarboxylic acid (TCA) cycle and mitochondrial electron transport chain (ETC.) complexes (Lill, 2009). Meanwhile, copper ions can allosterically regulate the activity and function of key metabolic enzymes such as pyruvate dehydrogenase (PHD). The dynamic equilibrium and metabolic flexibility between OXPHOS and aerobic glycolysis mediated by mitochondria are the critical foundation for cisplatin resistance in ovarian cancer (Dar et al., 2017). Specifically, Tumor cells orchestrate these two energy metabolic pathways and integrate metal ion-dependent redox homeostasis to establish a defensive system against cisplatin-induced cytotoxicity stress.

#### 2.1.2 Lipid metabolism

Lipid metabolic reprogramming is a key adaptive mechanism underlying cisplatin resistance in ovarian cancer. Resistant cells markedly enhance *de novo* lipogenesis by upregulating the expression of rate-limiting enzymes such as ATP-citrate lyase (ACLY) (Wei et al., 2021), acetyl-CoA carboxylase (ACC) (Li et al., 2013), and fatty acid synthase (FASN) (Jiang et al., 2014). This provides essential building blocks for membrane structure, lipid raft formation, signaling molecule generation, and the assembly of lipid droplets—key energy-storage organelles. Consequently, these changes effectively counteract cisplatin-induced membrane damage and maintain mitochondrial membrane integrity. Simultaneously, ovarian cancer cells upregulate lipid scavenger receptors (e.g., CD36) to extensively take up exogenous lipids, compensating for insufficient endogenous synthesis. Such alterations have been shown to promote tumor invasion and metastasis (Ladanyi et al., 2018). Lipid droplets formed through fatty acid synthesis and uptake not only supply membrane-building materials in response to oxidative and chemotherapeutic stress but also serve as energy reservoirs. Via lipolysis, stored fatty acids are released and enter mitochondria for β-oxidation, thereby supplying energy to support cell survival (Koizume et al., 2024). Similar to aerobic glucose oxidation, this process relies on OXPHOS and is thus influenced by iron and copper ion homeostasis. Furthermore, enhanced mitochondrial FAO not only generates substantial ATP to help cells cope with energy crisis and oxidative stress (Ma et al., 2018) but may also support DNA damage repair and the efficient operation of antioxidant defense systems involving copper and iron ions through the production of reducing equivalents such as NADH. Collectively, these adaptations strengthen cellular drug-resistance capacity through multiple integrated pathways (Cantó et al., 2015).

#### 2.1.3 Amino acid metabolism

In addition to glucose and lipid metabolism, ovarian cancer cells also coordinately reprogram amino acid metabolism—such as that of glutamine and serine—through multiple pathways to sustain survival and confer resistance to platinum-based drugs. In ovarian cancer cells, enhanced glutamine metabolism not only replenishes TCA cycle intermediates to supply energy and biosynthetic precursors in mitochondria, but also promotes glutathione synthesis, thereby alleviating oxidative stress (Fasoulakis et al., 2023); serine/glycine-driven one-carbon metabolism supports nucleotide synthesis, facilitates DNA repair, and contributes NADPH and folate to maintain redox homeostasis in both mitochondria and the cytosol (Wallace-Povirk et al., 2024); tryptophan metabolism *via* the kynurenine pathway elevates NAD^+^ levels, thereby supporting mitochondrial energy metabolism and scavenging reactive oxygen species; concurrently, arginine auxotrophy is associated with the epigenetic silencing of argininosuccinate synthase 1 (ASS1) (Nicholson et al., 2009); cysteine, as an antioxidant component, enhances cellular detoxification capacity; meanwhile, the high consumption of methionine is positively correlated with drug resistance. Nevertheless, its potential for overcoming cisplatin resistance in ovarian cancer and improving survival prognosis remains unclear (Cloven et al., 2000). It is noteworthy that the aforementioned reprogramming of amino acid metabolism is also associated with the homeostatic regulation of metal ions such as copper and iron. Under oxidative stress induced by platinum-based drugs, the glutathione synthesis system supported by glutamine metabolism needs to be coordinated with the homeostasis of iron, copper, and other ions in cells—the redox cycling of these ions within cells is closely associated with intracellular glutathione levels (Hatori et al., 2016; Xue et al., 2025). NADPH generated from serine/glycine metabolism and NAD^+^ produced *via* the tryptophan-kynurenine pathway may likewise influence mitochondrial ion valence, distribution, and activity by modulating the pool of reducing equivalents. This connection suggests that in ovarian cancer cells, the amino acid metabolic reprogramming associated with drug resistance involves multiple regulatory interactions with metal ion homeostasis that remain to be fully elucidated.

### 2.2 Impact of mitochondrial metal ion homeostasis on cancer cell drug resistance

**FIGURE 2:**
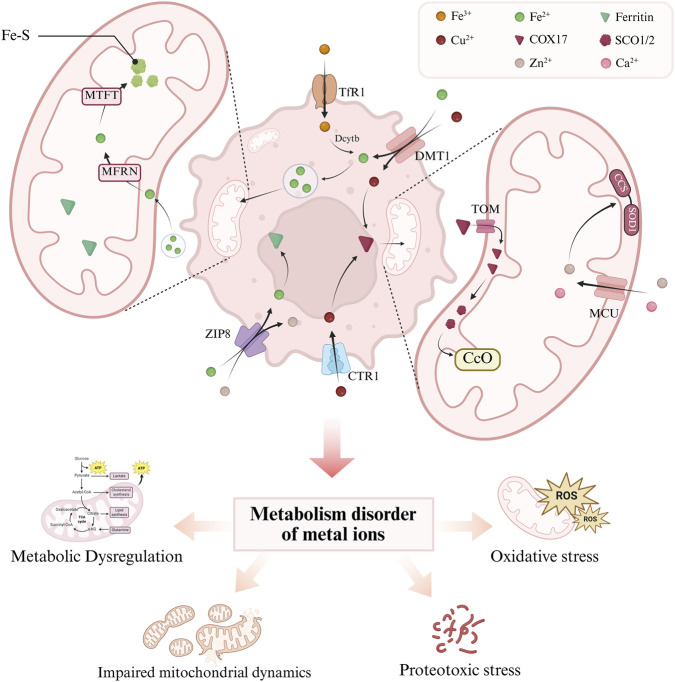
Association between mitochondrial ion homeostasis and cell death. Fe^3+^ enters cells *via* TFR1 and is reduced to Fe^2+^ by enzymes such as Dcytb. Fe^2+^ participates in iron-sulfur cluster biosynthesis, while mitochondrial iron transporters and iron regulatory proteins regulate iron trafficking and distribution within the cell and mitochondria. Cu^2+^ is imported into cells through CTR1 and is subsequently delivered by chaperones such as COX17 and COA1/2, thereby affecting the function of COX in mitochondria. Zn^2+^ transport involves proteins such as zinc transporter ZIP8, while the MCU controls Ca^2+^ entry into mitochondria. Dysregulation of these metal ions leads to metabolic disturbances, including aberrant metabolic pathways, oxidative stress (excessive ROS production and impaired antioxidant systems such as superoxide dismutase 1), impaired mitochondrial dynamics (abnormal morphology and fission-fusion balance), and proteotoxic stress (accumulation of misfolded proteins). These disruptions collectively drive the progression of cell death.(Figure was created with BioRender.com). Abbreviations: Fe-S, Iron-sulfur cluster; MTFT, Mitochondrial iron transporter; MFRN, Mitoferrin; TFR1, Transferrin receptor 1; Dcytb, Duodenal cytochrome b; DMT1, Divalent metal transporter 1; TOM, Translocase of outer mitochondrial membrane; COX17, Cytochrome c oxidase assembly factor 17; COA1/2, Cytochrome c oxidase assembly factors 1/2; SCO1/2, Synthesis of cytochrome c oxidase 1/2; ZIP8, Zrt-/Irt-like Protein 8; CTR1, Copper transporter 1; CcO, Cytochrome c oxidase; SOD1, Superoxide dismutase 1; MCU, Mitochondrial calcium uniporter.

The homeostasis of various metal ions in mitochondria, such as Fe^2+^/Fe^3+^, Cu^2+^, and Ca^2+^, serves not only as cofactors for key enzymes but also regulates respiratory chain function, thereby directly impacting cellular energy metabolism and redox balance (Figure 2). This regulation is a core determinant of cellular fate. These metal ions form a dynamic regulatory network that collectively maintains mitochondrial structural integrity and functional stability and plays a crucial role in mitochondrial biochemistry (Wang X. et al., 2021). They exist within mitochondria as labile “pools”, and the specific composition and function of such ion pools remain unclear (Lindahl and Moore, 2016).

Within mitochondria, copper ions play two central roles. First, they serve as essential cofactor in the catalytic center of cytochrome c oxidase (COX, directly driving the terminal reaction of the electron transport chain. Concurrently, copper is a critical component of antioxidant enzymes such as copper-zinc superoxide dismutase (SOD1), which localizes to the mitochondrial intermembrane space and scavenges superoxide anion radicals. Second, by modulating the activity of these enzymes, copper ions indirectly influence the overall efficiency and energy output of OXPHOS. Iron ions are mainly transported into the mitochondrial matrix through specific carrier Ferritin, where they serve as core components for the synthesis of iron-sulfur clusters and heme. Iron-sulfur clusters are indispensable cofactors for respiratory chain complexes I, II, and III, while heme is an essential prosthetic group of mitochondrial electron transport chain complexes III and IV (Vercellino and Sazanov, 2022), making it crucial for maintaining mitochondrial membrane potential and ATP synthesis.

Therefore, through the precise division of labor and synergistic actions described above, copper and iron ions collectively constitute a core regulatory network in the mitochondrial metal ion homeostasis system. This network directly governs electron transport chain efficiency, influences the balance between ROS generation and scavenging, and modulates cellular energy metabolism. Under cisplatin exposure, alterations occur in this mitochondrial metal network. Intracellularly, cisplatin disrupts the function of the copper chaperone protein Atox1, impeding its ability to deliver copper to ATP7B—a key transporter in copper efflux pathways—and thereby disturbing the normal distribution and utilization of cellular copper (Palm et al., 2011). Consequently, the resulting intracellular copper accumulation can promote lipid peroxidation *via* Fenton-like reactions, leading to structural damage of cellular components (Gaetke et al., 2003; Tsang et al., 2021). In normal cells, metal ions such as copper and iron act as essential cofactors for numerous key proteins, and their homeostatic metabolism underpins fundamental cellular processes; In ovarian cancer cells, however, metabolic reprogramming and crosstalk with metal ions drive copper and iron metabolism and function away from the normal physiological state (Alarcón-Veleiro et al., 2025; Yaman et al., 2007); In drug-resistant ovarian cancer cells, the metabolism and transport of metal ions such as copper and iron also undergo abnormal or adaptive changes under cisplatin treatment, alongside alterations in metabolism and redox homeostasis. Therefore, further analysis of how mitochondrial metal ion homeostasis influences metabolic preferences and redox systems in ovarian cancer cells may help identify novel strategies for overcoming cisplatin resistance (Table 1).

**TABLE 1:** Key pathways and molecules involved in cisplatin resistance, cuproptosis, and ferroptosis.

Category	Key molecules/Pathways	Primary role in drug resistance/Cell death	Evidence in ovarian cancer	References
Cisplatin Resistance(pre-target)	CTR1↓, ATP7A/B↑	Reduced drug influx; increased efflux and inactivation	Documented in ovarian cancer cell lines; limited clinical validation	Galluzzi et al. (2014), Yoshida et al. (2013), Lukanović et al. (2020), Li et al. (2017), Criscuolo et al. (2022), Kawahara et al. (2019)
Cisplatin Resistance(on-target)	ERCC1 (NER)↑, MMR deficiency	Enhanced DNA repair; failure to recognize platinum-DNA adducts	Established in ovarian cancer models and patient cohorts	Zhang et al. (2021), Helleman et al. (2006), Press et al. (2008)
Cisplatin Resistance(post-target)	p53↓, Bcl-2↑, IAPs↑	Apoptotic evasion	Documented in ovarian cancer tissues and cell lines	Fraser et al. (2008), Yuan et al. (2020), McCabe et al. (2014)
Cisplatin resistance (off-target)	EGFR/ErbB2↑, DYRK1B↑	Compensatory survival signaling; antioxidant responses	Observed in resistant ovarian cancer cells	Yang et al. (2020), Gao et al. (2012)
Cuproptosis	FDX1, LIAS, DLAT, DLST	Copper-induced aggregation of lipoylated TCA enzymes; metabolic collapse	Limited direct evidence in ovarian cancer; mainly derived from general cancer models	(Tian et al., 2023; Tsvetkov et al., 2022) Zulkifli et al. (2023), Lin et al. (2024), Dreishpoon et al. (2023)
Ferroptosis	GPX4, SLC7A11, ACSL4, FSP1	Iron-dependent lipid peroxidation	Partially validated in ovarian cancer cells; some components extrapolated from other models	Zhou et al. (2024b), Doll et al. (2017), Yang et al. (2014), Wang et al. (2024), Li et al. (2021), Ma et al. (2024b)
Shared regulatory nodes	Fe-S clusters, GSH, NADPH, ROS, mitochondrial, ETC	Redox balance; antioxidant capacity	Derived from general cell biology; direct evidence in ovarian cancer is lacking	Lill, 2009; Hatori et al. (2016), Gaetke et al. (2003), Tsang et al. (2021)

Because copper and iron ions are central to mitochondrial redox balance, their disruption does more than just alter metabolism—it also determines whether cancer cells undergo specific forms of cell death. In the sections that follow, we examine how imbalances in copper and iron trigger cuproptosis and ferroptosis, and discuss the potential of targeting these pathways to reverse cisplatin resistance.

## 3 Cuproptosis: the “metabolic trap” for cisplatin-resistant cells

Tumor cells exhibit diverse modes of cell death. Beyond classical apoptosis, autophagy, and necrosis, emerging forms of programmed cell death such as cuproptosis and ferroptosis have attracted extensive attention in recent years (Kci et al., 2024). These modes of cell death differ in their triggering mechanisms and phenotypic characteristics, yet all are closely linked to cellular metabolic states, such as redox balance, fatty acid metabolism, and glucose metabolism. Mechanistically, cuproptosis and ferroptosis are intimately associated with mitochondria metal ion homeostasis. In particular, dysregulation of copper homeostasis can induce cell death through distinct mechanisms: excess copper ions disrupt mitochondrial protein lipoylation and directly trigger oxidative stress in both mitochondria and the cytoplasm; conversely, copper deficiency impairs the activity of key enzymes involved in glucose and lipid metabolism and leads to dysfunction of the electron transport chain. Since drug-resistant tumor cells frequently undergo metabolic reprogramming and become highly dependent on specific metabolic pathways, metabolism-linked death pathways such as cuproptosis may represent a potential vulnerability in these cells.

### 3.1 The essence of cuproptosis—metabolic cell death

**FIGURE 3:**
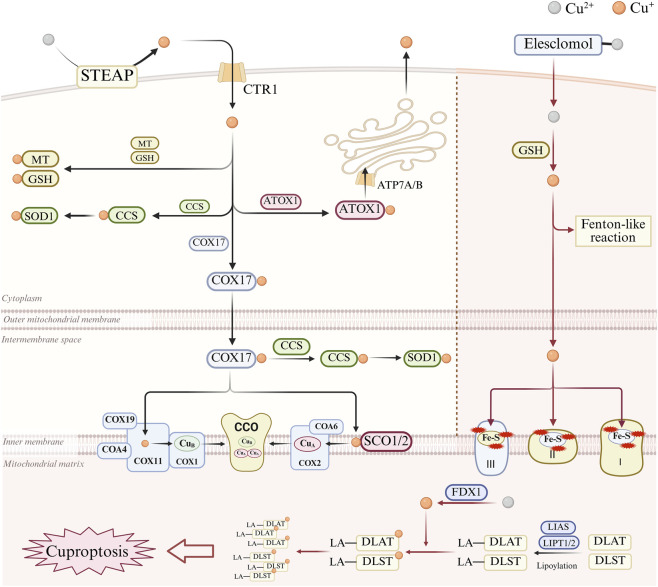
Copper metabolism and cuproptosis. Normal Cellular Copper Metabolism: on the cell membrane, Cu^2+^ is reduced to Cu^+^ by the STEAP family proteins and imported into the cell *via* CTR1. Within the cytosol, Cu^+^ is distributed through distinct pathways: A portion is delivered by ATOX1 to the Golgi-localized transporters ATP7A or ATP7B, mediating copper efflux; Another fraction binds to MT or GSH; Cu^+^ is also transferred by CCS to activate SOD1; Additionally, Cu^+^ is delivered by COX17 and other assembly factors for incorporation into mitochondrial COX. Dysregulation Induced by Copper Ionophores: when copper ionophores such as elesclomol transport Cu^2+^ into cells, Cu^2+^ is reduced (e.g., by GSH) and can catalyze the conversion of hydrogen peroxide to hydroxyl radicals *via* Fenton-like reactions, leading to structural damage. More critically, free Cu^+^ entering mitochondria *via* ion transporters disrupts Fe-S clusters through a dual mechanism: As a potent reductant, Cu^+^ directly attacks the Fe-S core, reducing Fe^3+^ to unstable Fe^2+^, causing iron displacement and cluster disintegration. Concurrently, Cu^+^ drives hydroxyl radical generation through Fenton-like reactions, resulting in irreversible oxidative damage to Fe-S clusters. Together, these pathways completely abolish electron transfer function in respiratory chain complexes. Furthermore, Cu^2+^—facilitated by FDX1 and involving LIAS and LIPT1/2—binds to the lipoylamide groups of lipoylated enzymes such as DLAT and DLST. This binding induces oligomerization of lipoylated DLAT and DLST, ultimately triggering cuproptosis. (Figure was created with BioRender.com). Abbreviations: STEAP, Six-transmembrane epithelial antigen of prostate; ATOX1, Antioxidant protein 1; ATP7B, Copper transport ATPases 7B; CCS, Copper chaperone for superoxide dismutase; SOD1, Superoxide dismutase 1; SCO1/2, Synthesis of cytochrome c oxidase 1/2; SLC25A3, Solute carrier family 25 member 3; FDX1, Ferredoxin 1; LIAS, Lipoic acid synthase; DLAT, Dihydrolipoamide acetyltransferase; DLST, Dihydrolipoamide succinyltransferase; LA, Lipoic acid; CCO, Cytochrome c oxidase.

Cuproptosis, a novel form of regulated cell death discovered and named by Tsvetkov et al., in 2022, is a metabolism-dependent programmed cell death pathway that plays a key role in ovarian cancer progression, chemotherapy resistance, and the tumor immune microenvironment (Tian et al., 2023; Tsvetkov et al., 2022). A defining feature of cuproptosis is its direct induction by copper ions (Cu^2+^/Cu^+^), which target and disrupt the assembly of mitochondrial respiratory chain complexes. Moreover, copper ions can impair iron-sulfur clusters within these complexes through multiple mechanisms, thereby interfering with cellular OXPHOS. Consequently, metabolically active tumor cells in ovarian cancer tissues that rely heavily on OXPHOS for energy production exhibit heightened sensitivity to cuproptosis (Tsvetkov et al., 2022). The occurrence of cuproptosis is not simply due to copper excess but directly results from disruption of the finely regulated intracellular copper homeostasis system (Figure 3). This offers a new theoretical foundation and therapeutic direction for targeting cancer metabolism, although the exact role of cuproptosis in ovarian cancer is still not well understood.

#### 3.1.1 Copper ion accumulation-mediated cell death

Copper ions are essential cofactors in all living organisms, and their intracellular fate is regulated by a complex homeostatic system composed of transmembrane transporters and copper chaperone proteins. Studies have shown that structurally diverse copper ionophores, such as elesclomol and disulfiram (DSF), can elicit consistent cytotoxic effects in tumor cells (Hunsaker and Franz, 2019; Cen et al., 2004; Zheng et al., 2022). This effect can be reversed by copper chelators such as tetrathiomolybdate (TTM). In contrast, pharmacological inhibitors targeting various cell death pathways—including necrostatin-1 and N-acetylcysteine—have been found to be ineffective in suppressing copper-induced cell death. This finding underscores that cuproptosis represents a distinct form of regulated cell death governed by a unique regulatory mechanism and network that differs from all other known programmed cell death pathways (Alvarez et al., 2010).

Prior to cellular uptake, Cu^2+^ is reduced to Cu^+^ by six-transmembrane epithelial antigen of prostate (STEAP) family proteins (Ohgami et al., 2006) and transported into the cytoplasm primarily *via* the copper uptake protein CTR1 (SLC31A1). Once inside the cell, copper ions are precisely delivered by a series of specific chaperone proteins: ATOX1 transfers copper to the ATP7A/B pumps in the Golgi apparatus, which subsequently export copper from the cell or load it into copper-dependent enzymes (Suwara and Hartman, 2025); CCS delivers copper to superoxide dismutase (SOD1) to maintain ROS homeostasis (Boyd et al., 2020); and cytochrome c oxidase 17 (COX17) transports copper into mitochondria for the assembly of COX (Cobine et al., 2006; Liu et al., 2014). In addition, MT and GSH serve as crucial intracellular copper-chelating buffer systems, effectively preventing toxicity caused by excessive free copper ion concentrations (Calvo et al., 2017). Copper ionophores disrupt this finely tuned regulatory network by mediating the direct entry of Cu^2+^ into cells, leading to abnormal copper accumulation within mitochondria and thereby triggering downstream death signaling.

#### 3.1.2 Mitochondrial dependence of copper ion cytotoxicity

Mitochondria, the central hub for cellular energy metabolism and redox balance, directly determine a cell’s susceptibility to, and adaptive response to, copper toxicity. The occurrence of cuproptosis depends on functional TCA cycling and aerobic respiration (Qi and Zhu, 2023). Cells reliant on mitochondrial OXPHOS are far more sensitive to copper ionophores than those dependent on glycolysis, as reported in lung cancer and other tumor models (Tian et al., 2023; Tsvetkov et al., 2022); whether this applies to ovarian cancer cells remains to be determined. Multiple studies indicate that cuproptosis is closely associated with excessive copper accumulation, abnormal modification of key mitochondrial enzymes, and disruption of iron-sulfur cluster integrity. Thus, mitochondrial dysfunction constitutes a critical component of copper-induced cell death (Horn and Barrientos, 2008).

Ferredoxin 1 (FDX1), located in the mitochondrial matrix, is a central regulator in cuproptosis, linking lipoylation and copper metabolism through a dual mechanism to drive the process. On one hand, FDX1 acts as a reductase that directly reduces Cu^2+^ to Cu^+^, facilitating the release of copper ions from chaperone proteins and enhancing their binding affinity for lipoylated proteins (Zulkifli et al., 2023). On the other hand, FDX1 directly interacts with lipoic acid synthase (LIAS), promoting its maturation and activity, thereby regulating protein lipoylation levels and ultimately inducing cell death (Lin et al., 2024; Dreishpoon et al., 2023). Copper ions form high-affinity bonds with cysteine residues in lipoylated proteins such as dihydrolipoamide acetyltransferase (DLAT), pyruvate dehydrogenase complex component X (PDHX), and dihydrolipoamide succinyltransferase (DLST). This binding triggers oligomerization and functional loss of these proteins, leading to inactivation of key TCA cycle-related enzymes, including the pyruvate dehydrogenase complex (PDC) and α-ketoglutarate dehydrogenase complex (α-KGDHC). These disruptions not only directly interrupt cellular energy flux but also deplete NADPH, weaken antioxidant defenses, and promote electron leakage from the electron transport chain. Together, these effects generate oxidative stress beyond cellular compensatory capacity, ultimately driving cell death (Rowland et al., 2018; Giles et al., 2003).

Another driving force of cuproptosis is the direct impact of free copper ions on the activity and function of mitochondrial electron transport chain complexes (Tsvetkov et al., 2022). In the mitochondrion—an environment rich in oxygen and reducing equivalents—Cu^+^ acts as a potent pro-oxidant. First, Cu^+^ displaces iron ions within the iron-sulfur clusters (Fe-S clusters) of respiratory chain complexes I, II, and III. This alters the atomic spacing, bond angles, and overall spatial arrangement of the clusters, disrupting electron transfer, directly inhibiting respiratory chain function, and causing electron leakage (Tian et al., 2023; Cai et al., 2017).The leaked electrons react with oxygen to generate superoxide anions (O_2_•^-^), which are subsequently dismutated into hydrogen peroxide (H_2_O_2_). Cu^+^ then catalyzes the conversion of H_2_O_2_ into highly destructive hydroxyl radicals (•OH) *via* the Fenton reaction, leading to a sharp increase in oxidative stress (G et al., 2003; Tsang et al., 2021). Concurrently, the cell consumes large amounts of core antioxidants such as GSH in an attempt to counteract copper toxicity, further depleting its reductive capacity. This ultimately establishes a vicious cycle that irreversibly impairs mitochondrial function. The displacement of iron from Fe-S clusters by copper has been primarily demonstrated in biochemical and non-ovarian cancer models; whether this mechanism operates efficiently in ovarian cancer cells remains to be directly tested.

Overall, cuproptosis can be fundamentally regarded as a mitochondrion-dependent, metabolically driven form of cell death. Its underlying mechanisms highlight the intricate interdependence and mutual regulation between metal homeostasis and cellular metabolic networks, offering important insights into the development of tumor drug resistance.

### 3.2 Metabolic association between cuproptosis and cisplatin resistance in ovarian cancer

Altered copper homeostasis in ovarian cancer constitutes a critical component of metabolic reprogramming under pharmacological stress and has emerged as a key metabolic feature influencing disease progression and chemotherapeutic efficacy. Multiple clinical studies have shown that serum and tumor tissue copper levels are significantly elevated in ovarian cancer patients compared with healthy individuals, and high copper levels are independently associated with advanced clinical stage and poor prognosis (Yaman et al., 2007). Such sustained intracellular copper accumulation supports copper-dependent biological processes required for malignant behaviors such as rapid proliferation, angiogenesis, and metastasis—for instance, by promoting epithelial-mesenchymal transition (EMT) and metastasis through the ATOX1-ATP7A-LOX signaling axis (Chen and Liu, 2024). However, the metabolic changes induced by abnormal copper accumulation can also render ovarian cancer cells vulnerable to a “metabolic trap,” wherein the very mechanisms that support survival simultaneously increase their susceptibility to cuproptosis and push them toward cell death.

Interestingly, there is mechanistic overlap and functional coupling between dysregulated copper metabolism and cisplatin resistance in ovarian cancer. The membrane transporter CTR1 (SLC31A1), responsible for copper uptake, also mediates the intracellular influx of cisplatin. Downregulation of CTR1 observed in cisplatin-resistant ovarian cancer cells may consequently impair copper transport (Holzer et al., 2004). A clinical study of Chinese epithelial ovarian cancer patients receiving platinum-based chemotherapy found that the CTR1 rs10981694 polymorphism is associated with carboplatin resistance, with G allele carriers showing a higher risk of resistance (OR = 4.00, 95% CI: 1.309–12.23, p < 0.01) (Li et al., 2017). Meanwhile, ATP7A and ATP7B, which mediate copper efflux to maintain metal homeostasis, are also widely involved in cisplatin export (Guo et al., 2023; Lasorsa et al., 2019). Studies have shown that the copper transporter ATP7A acts as a negative regulator of cisplatin sensitivity. Elevated ATP7A expression in ovarian cancer cells directly confers resistance to cisplatin, carboplatin, and oxaliplatin. The mechanism involves ATP7A sequestering platinum drugs into intracellular vesicular compartments, preventing access to nuclear DNA targets and thereby blocking cytotoxicity. Notably, while platinum drugs do not efficiently activate the ATP7A efflux function as copper does, ATP7A still confers protection and ultimately drives drug resistance (Samimi et al., 2004).However, emerging research suggests that elesclomol may activate copper transporters, simultaneously promoting the influx of both copper and cisplatin, thereby increasing intracellular cisplatin concentration and enhancing its toxicity against ovarian cancer cells. Pretreatment of ovarian cancer cells with low-dose elesclomol-Cu complexes significantly reduces the half-maximal inhibitory concentration (IC_50_) of subsequent cisplatin treatment. In tumor-bearing mouse models, this combination not only markedly enhances antitumor efficacy but also alleviates cisplatin-induced weight loss and renal toxicity (Zou et al., 2025). Thus, cuproptosis-targeted therapy and cisplatin chemotherapy exhibit synergistic effects, suggesting a potential to reverse cancer cell resistance by interfering with shared copper metabolic pathways while demonstrating favorable safety. These findings, however, are based on preclinical models and await further validation. Although these findings suggest a link between dysregulated copper metabolism and cisplatin resistance in ovarian cancer, several questions remain unresolved. For instance, whether artificially induced copper overload in ovarian cancer cells upregulates protective proteins such as ATP7A, and the precise mechanisms by which ovarian cancer cells develop cisplatin resistance through copper transport systems and metabolic processes, have yet to be fully elucidated.

Beyond shared transport proteins, the interplay between copper metabolism and cisplatin action is also reflected in their impact on cancer cell metabolism. Copper overload directly inhibits key TCA cycle enzymes such as PDC and α-KGDHC, leading to cycle disruption and severe depletion of ATP and NADPH in non-ovarian cancer models (Tennant et al., 2010; Sullivan et al., 2015; Xiao et al., 2018). The resulting energy deficit impairs the ATP-dependent NER system, hindering the effective removal of cisplatin-induced DNA adducts (Tennant et al., 2010). Simultaneously, the depletion of TCA cycle intermediates—such as aspartate and succinate—compromises the substrate pool required for nucleotide biosynthesis, causing insufficient dNTP supply and further weakening DNA damage repair capacity (Sullivan et al., 2015). Moreover, NADPH exhaustion collapses the glutathione reductase system (Xiao et al., 2018), leaving cells with inadequate reductive power to counteract the oxidative stress jointly triggered by cuproptosis and cisplatin and thereby exacerbating macromolecular damage. The widespread disruption of energy metabolism and redox homeostasis induced by cuproptosis interferes with the formation and regulation of tumor cell drug resistance at the metabolic level. Whether these metabolic disruptions occur similarly in cisplatin-resistant ovarian cancer cells requires direct investigation. Nevertheless, the specific connections and cross-regulatory mechanisms between copper ions and platinum drugs in terms of intracellular transport and metabolism remain incompletely understood. Exploiting cuproptosis induction to reverse cisplatin resistance represents an intervention strategy that still requires in-depth investigation.

### 3.3 Application potential of cuproptosis in the prevention and treatment of ovarian cancer

Cuproptosis demonstrates translational promise in prognostic prediction and targeted therapy for ovarian cancer. To explore its clinical applicability, this section focuses on two strategies: first, the development of precise prognostic models to guide clinical decision-making; and second, the design of novel therapeutic approaches targeting cuproptosis to overcome drug resistance.

#### 3.3.1 lncRNA-related molecular typing and prognostic prediction of ovarian cancer

Prognostic prediction models based on molecular signatures associated with cuproptosis represent the first step toward personalized treatment. Long non-coding RNAs (lncRNAs) play important roles in the regulatory network of cuproptosis in cancer by modulating copper metabolism, mitochondrial function, and key signaling pathways (Wang et al., 2023; Jiang N. et al., 2021). On one hand, specific lncRNAs can directly interfere with copper ion homeostasis by upregulating or downregulating the expression of critical copper transporters through epigenetic modifications, transcriptional regulation, or post-transcriptional mechanisms (Guo et al., 2024). This regulation directly determines net intracellular copper accumulation, creating the preconditions for cuproptosis. On the other hand, numerous oncogenic or tumor-suppressive lncRNAs—such as MALAT1 and H19—crosstalk with key signaling pathways including PI3K/AKT, Wnt/β-catenin, and p53, thereby modulating the balance between apoptosis and survival and indirectly shaping the cellular response to cuproptosis (Baba et al., 2023; Riquelme et al., 2023; Lin et al., 2020; Zhang et al., 2020; Lin et al., 2019). Using bioinformatics approaches, researchers have screened public databases such as TCGA to identify genes closely linked to cuproptosis (CRGs, e.g., FDX1 and LIAS) or cuproptosis-related lncRNAs (CRLs), successfully constructing several risk-scoring models (e.g., CuRScore) (Yang et al., 2024; Bhat et al., 2024).These models essentially quantify tumor cuproptosis activity. Interestingly, this metric shows a strong correlation with the tumor immune microenvironment. Immune microenvironment analysis of risk subgroups reveals that low-risk tumors (with high cuproptosis activity) often display a “hot tumor” phenotype characterized by an immunologically active state, including higher infiltration of CD8^+^ T cells and M1-like macrophages, as well as elevated expression of immune checkpoints such as PD-L1 and CTLA-4. This suggests that low-risk patients may be more suitable for immune checkpoint inhibitor (ICI) therapy, a notion supported by database analyses (e.g., the IMvigor210 cohort) (Zhou M. et al., 2024).Conversely, high-risk tumors (with low cuproptosis activity) predominantly exhibit an immunosuppressive phenotype, marked by increased infiltration of regulatory T cells (Tregs) and M2-like macrophages, higher stromal scores, and elevated TIDE scores—all indicative of poorer responses to immunotherapy. Thus, beyond being a form of programmed cell death, cuproptosis-related lncRNAs can serve as integrated biomarkers for predicting patient prognosis, chemotherapy response, and immunotherapy efficacy. These models enable stratification of ovarian cancer patients into distinct risk subgroups, providing insights into prognosis and sensitivity to conventional platinum-based chemotherapy as well as targeted agents such as poly(ADP-ribose) polymerase (PARP) inhibitors. Such stratification offers valuable reference information for guiding subsequent individualized therapeutic strategies.

#### 3.3.2 Cuproptosis-targeted therapeutic strategies for ovarian cancer

The therapeutic potential of cuproptosis in ovarian cancer management lies in its unique killing mechanism, which specifically targets the active mitochondrial metabolism and dysregulated copper homeostasis characteristic of chemotherapy-resistant tumors. In colorectal cancer models, elesclomol selectively delivers divalent copper ions into cells, triggering intense oxidative stress and inducing degradation of the copper transporter ATP7A (Gao et al., 2021). This blocks copper efflux pathways, leads to overload of free copper ions in mitochondria, and ultimately triggers cuproptosis by directly targeting lipoylated proteins in the tricarboxylic acid cycle. Notably, elesclomol exhibits significantly superior cytotoxicity against ovarian cancer stem-like tumor-initiating cells (TICs) compared with conventional drugs, offering a promising new direction for addressing clinical challenges such as disease recurrence and platinum resistance (Harrington et al., 2020).

Second, cuproptosis-based strategies can synergize with existing therapies to reverse drug resistance. DSF demonstrates multifaceted value that extends beyond its intrinsic toxicity (Caminear et al., 2022). The DSF/Cu complex acts as an effective cuproptosis inducer by depleting iron-sulfur cluster proteins and, *via* the p97-NPL4-UFD1 pathway, triggering endoplasmic reticulum stress-mediated cell death independent of apoptosis. Simultaneously, DSF potently inhibits the function of multiple drug efflux transporters, including P-glycoprotein, thereby increasing intracellular accumulation of platinum drugs and enhancing DNA-adduct formation, which promotes tumor cell death (Guo et al., 2023; McMahon et al., 2020; Sauna et al., 2004).*In vitro* studies confirm that even when the doses of DSF and cisplatin are reduced by a hundredfold, their combination still induces substantial cell death in resistant tumor cells. This “synergistic efficacy with reduced toxicity” further underscores the clinical translational potential of cuproptosis inducers such as DSF (Guo et al., 2022).

Finally, to overcome the potential off-target toxicity of traditional copper ionophores and enable tumor-specific delivery and precise induction of cuproptosis, the integration of nanotechnology and novel copper-based materials has become a focus of current research. Traditional disulfiram prodrug (DQ) co-loaded with copper in liposomes can be activated by high levels of reactive oxygen species in the tumor region, generating highly cytotoxic DDC-Cu complexes *in situ*. This not only efficiently triggers cuproptosis but also induces immunogenic cell death, creating opportunities for combination immunotherapy (Kang et al., 2023; Kang et al., 2021). Compared with such liposomal systems, a cuproptosis nanodrug named Lipo@CP@DQ NPs loads copper peroxide (CP), which rapidly dissociates in the acidic tumor microenvironment and initiates a self-amplifying cascade. The released copper ions directly induce cuproptosis while also catalyzing the generation of hydroxyl radicals from self-supplied H_2_O_2_. Concurrently, DQ decomposes into diethyldithiocarbamate (DTC), which chelates copper to form the anticancer compound Cu(DTC)_2_, and quinone methide (QM), which depletes GSH to amplify oxidative stress. This triple-synergy mechanism has been demonstrated in a triple-negative breast cancer (TNBC) mouse model (Yan et al., 2025). This triple-synergy mechanism—combining cuproptosis, *in situ* chemotherapy, and enhanced oxidative stress—effectively suppresses postoperative tumor recurrence and metastasis. Furthermore, a GSH-responsive smart nanoplatform, GOx@[Cu(tz)], releases glucose oxidase (GOx), which is activated within cancer cells characterized by high glucose consumption and elevated GSH levels. This depletes both glucose and GSH, creating metabolic vulnerabilities conducive to cuproptosis while simultaneously inducing starvation therapy and significantly amplifying the cuproptosis effect. The H_2_O_2_ generated during glucose oxidation further activates the material’s type-I photodynamic therapeutic efficacy, as reported in bladder cancer cells (Xu et al., 2022). Whether these nanoplatforms are effective in ovarian cancer remains to be investigated.

The application of cuproptosis in ovarian cancer prevention and treatment constitutes a multilayered and logically coherent integrated system: it exploits the metabolic characteristics of cancer cells for tumor elimination, synergizes with existing therapies to overcome resistance, and gains precision through nanotechnology. Nevertheless, most of the supporting evidence comes from non-ovarian cancer models. Whether this paradigm applies to cisplatin-resistant ovarian cancer remains to be validated in ovarian-cancer-specific studies.

## 4 Ferroptosis: an oxidative stress-driven death switch in ovarian cancer cells

### 4.1 Iron metabolism and ferroptosis

**FIGURE 4:**
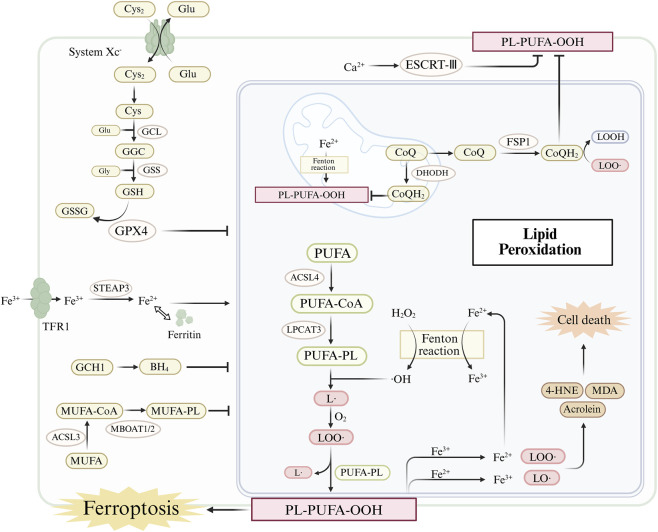
Molecular mechanism and regulatory pathway of ferroptosis. Ferroptosis is fundamentally driven by a self-amplifying cycle of lipid peroxidation, initiated when PUFAs are activated by ACSL4 and incorporated into membrane phospholipids by LPCAT3, forming PUFA-PL. The peroxidation cascade begins with the formation of a lipid radical (L•), which reacts with oxygen to generate a lipid peroxyl radical (LOO•). LOO• then abstracts a hydrogen atom from another PUFA-PL, producing a lipid hydroperoxide (LOOH) and a new L•, thereby propagating a chain reaction that accumulates PL-PUFA-OOH and ultimately leads to membrane disruption and cell death. Iron plays a central catalytic role in this process: Fe^3+^ is reduced to Fe^2+^ by PL-PUFA-OOH, and Fe^2+^ further fuels radical generation *via* Fenton reactions, while radicals such as LOO• continue to oxidize additional PUFA-PL and generate toxic secondary products like 4-HNE. Cellular defense against ferroptosis operates through multiple parallel pathways: the system Xc^−^ imports cystine for GSH synthesis, enabling GPX4 to reduce lipid hydroperoxides; iron homeostasis is regulated by TFR1 and ferritin; and molecules such as tetrahydrobiopterin (BH_4_) and MUFA-PL exert inhibitory effects. Moreover, the ESCRT-III-mediated membrane repair machinery provides an additional layer of protection by mitigating membrane damage inflicted by PL-PUFA-OOH. Together, these regulatory networks determine cellular susceptibility to ferroptosis, balancing peroxidative stress with adaptive resistance mechanisms. (Figure was created with BioRender.com). Abbreviations: System Xc^−^, Cystine/glutamate antiporter; Glu, Glutamate; Cys, Cysteine; GCL, Glutamate-cysteine ligase; GGC, γ-Glutamylcysteine; Gly, Glycine; GSS, Glutathione synthetase; GSH, Glutathione; GSSG, Oxidized glutathione; GPX4, Glutathione peroxidase 4; TFR1, Transferrin receptor 1; STEAP3, Six-transmembrane epithelial antigen of prostate 3; GCH1, GTP cyclohydrolase 1; BH_4_, Tetrahydrobiopterin; MUFA, Monounsaturated fatty acid; ACSL3, Acyl-CoA synthetase long-chain family member 3; MUFA-CoA, Monounsaturated Acyl-Coenzyme A; MBOAT1/2, Membrane-bound O-acyltransferase 1/2; MUFA-PL, Monounsaturated fatty acid-phospholipid; PUFA, Polyunsaturated fatty acid; ACSL4, Acyl-CoA synthetase long-chain family member 4; PUFA-CoA, Polyunsaturated Acyl-Coenzyme A; LPCAT3, Lysophosphatidylcholine acyltransferase 3; PUFA-PL, Polyunsaturated fatty acid-phospholipid; ・OH, Hydroxyl radical; LOO・, Lipid peroxyl radical; LO・, Lipid oxy radical; L・, Lipid radical; 4-HNE, 4-Hydroxynonenal; MDA, Malondialdehyde; Acrolein, Acrolein; CoQ, Coenzyme Q; DHODH, Dihydroorotate dehydrogenase; FSP1, Ferroptosis suppressor protein 1; CoQH_2_, Ubiquinol; PL-PUFA-OOH, Phospholipid-polyunsaturated fatty acid hydroperoxide; ESCRT-III, Endosomal sorting complex required for transport III.

Ferroptosis is an iron-dependent form of regulated cell death first proposed by Dixon et al., in 2012, is fundamentally driven by the accumulation of lipid peroxides (Zhou Q. et al., 2024). Its mechanism involves dysregulated intracellular iron metabolism, failure of antioxidant defense systems such as the GPX4 pathway, and excessive peroxidation of phospholipids containing polyunsaturated fatty acids. Notably, ferroptosis arises not only from iron overload but also from disruption of cellular iron homeostasis and redox balance (Figure 4). Current evidence suggests a close link between ferroptosis and cisplatin resistance in ovarian cancer; however, the underlying regulatory network and mechanistic details remain incompletely defined. Our current understanding of ferroptosis mechanisms is derived mainly from non-ovarian cancer models, and direct evidence in ovarian cancer is still relatively limited.

#### 4.1.1 Driving mechanism of ferroptosis

Ferroptosis relies on the excessive peroxidation of polyunsaturated fatty acids (PUFAs) within membrane phospholipids, a process primarily catalyzed by the coordinated action of acyl-CoA synthetase long-chain family member 4 (ACSL4) and lysophosphatidylcholine acyltransferase 3 (LPCAT3). ACSL4 first activates PUFAs to form acyl-CoA esters, which are then esterified and incorporated into membrane phospholipids such as phosphatidylethanolamine by LPCAT3, thereby providing structural substrates for lipid peroxidation (Doll et al., 2017; Dixon et al., 2015). These initial discoveries were made in non-ovarian cancer cell lines, however, ACSL4 expression has been confirmed in ovarian cancer cells and is functionally relevant, as discussed later in this section. On the other hand, the functional state of GPX4, a core component of the cellular antioxidant defense system, is critical for suppressing ferroptosis (Yang et al., 2014). Using GSH as a reductant, GPX4 specifically catalyzes the reduction of lipid hydroperoxides (LOOH) to their corresponding alcohols (LOH), thereby blocking the propagation of lipid radical chain reactions. GPX4 activity is highly dependent on GSH regeneration, whose synthesis requires cystine uptake mediated by the system Xc^−^ antiporter, which iscomposed of SLC7A11 and SLC3A2 subunits (Liu et al., 2021). Thus, inhibition of SLC7A11 by erastin or direct suppression of GPX4 by ras-selective lethal small molecule 3 (RSL3) leads to massive accumulation of lipid peroxides and ultimately induces ferroptosis (Sato et al., 2018). Furthermore, dysregulated iron metabolism plays a key role in ferroptosis. The transferrin receptor 1 (TFR1)-mediated endocytosis (Richardson and Ponka, 1997), NCOA4-mediated ferritinophagy (Gao et al., 2016), and STEAP3-mediated reduction of Fe^3+^ to redox-active Fe^2+^ collectively regulate the cellular labile iron pool (LIP). Fe^2+^ catalyzes the conversion of lipid peroxides into highly reactive lipid radicals *via* the Fenton reaction, accelerating chain propagation and amplification of lipid peroxidation, thereby promoting ferroptosis (Winterbourn, 1995).

#### 4.1.2 Synergistic regulation of ferroptosis by intracellular signaling pathways and immune microenvironment

In tumor cells, the induction and downstream effects of ferroptosis are jointly regulated by the intrinsic molecular features of cancer cells and the tumor microenvironment. At the intracellular level, tumor suppressor genes such as p53 promote iron-dependent lipid peroxidation and cell death by transcriptionally repressing SLC7A11, the light chain component of the cystine/glutamate antiporter, thereby reducing cystine uptake (Jiang et al., 2015). Similarly, BRCA1-associated deubiquitinase 1 (BAP1) enhances cellular sensitivity to ferroptosis by inhibiting SLC7A11 expression through histone deubiquitination (Zhang Y. et al., 2018).Conversely, oncogenic signaling pathways can counteract ferroptosis. Activation of the nuclear factor erythroid 2-related factor 2 (NRF2) pathway upregulates multiple antioxidant-related genes—including heme oxygenase-1 (HO-1), ferritin heavy chain 1 (FTH1), and NAD(P)H:quinone oxidoreductase 1 (NQO1)—which effectively neutralize lipid peroxidation products and suppress ferroptosis (Zhou Q. et al., 2024; Han et al., 2022). Additionally, the RAS-MAPK and mTOR pathways confer resistance to ferroptosis by modulating lipid metabolism and intracellular antioxidant capacity. Notably, within the tumor microenvironment (TME), ferroptosis and immune responses engage in close crosstalk. Tumor cells undergoing ferroptosis release damage-associated molecular patterns (DAMPs), which activate dendritic cells and enhance the antitumor function of CD8^+^ T cells, establishing an immunogenic cell death phenotype (Zhang et al., 2022). On the other hand, activated CD8^+^ T cells secrete IFN-γ, which downregulates SLC7A11 expression in tumor cells, further promoting iron-dependent cell death and forming a positive feedback loop in antitumor immunity (Kci et al., 2024; Zhou et al., 2022). The coordinated regulation of ferroptosis-related signaling pathways by intracellular factors, immune cells and cytokines offers a novel perspective on tumor-microenvironment interactions. Elucidating these mechanisms may contribute to more holistic therapeutic strategies targeting the tumor as a whole. In the following section, we will explore the potential of modulating ferroptosis from a metabolic standpoint to reverse drug resistance.

### 4.2 Metabolic association between ferroptosis and cisplatin resistance in ovarian cancer

In various cancers, including ovarian cancer, iron metabolism and ferroptosis have been shown to correlate with cisplatin resistance through redox metabolic pathways. Inducing ferroptosis can bypass apoptosis evasion and counteract resistance mechanisms such as enhanced antioxidant capacity and drug efflux, as shown in multiple cancer types) (Su et al., 2020). Cisplatin itself can initially trigger ferroptosis by inducing and ROS burst and mitochondrial dysfunction, thereby promoting lipid peroxidation and contributing to tumor cell killing. However, in acquired resistant tumor cells, activation of adaptive metabolic pathways concurrently suppresses ferroptosis. Enhancement of the GPX4-GSH antioxidant axis represents one of the most classic pre- and/or on-target chemoresistance mechanisms (Hangauer et al., 2017). As an essential cofactor for GPX4, GSH reduces toxic lipid peroxides, maintains redox homeostasis, and consequently diminishes cisplatin-induced ferroptosis sensitivity. Moreover, the tumor suppressor p53, a key regulator of ferroptosis, promotes ferroptosis in ovarian cancer by downregulating SLC7A11 expression (Han et al., 2024). Inactivation or mutation of the p53 pathway in resistant cells may therefore upregulate SLC7A11 and GPX4, leading to ferroptosis resistance. Consistent with this mechanism, a recent clinical cohort study of 192 epithelial ovarian cancer patients confirmed that high co-expression of SLC7A11 and GPX4 in tumor tissues is independently associated with platinum resistance (PFI <6 months) and predicts significantly shorter progression-free and overall survival (Wu et al., 2022). ACSL4 acts as a key positive regulator of ferroptosis. Cisplatin treatment induces iron accumulation and elevated lipid peroxidation in ovarian granulosa cells, accompanied by upregulation of ACSL4. Mechanistically, cisplatin activates transcription factor SP1, which directly binds to the promoter region of the ACSL4 gene (−207 to −197 bp) and enhances its transcription (Wang et al., 2024). ACSL4 activates long-chain PUFAs into acyl-CoA esters and incorporates them into membrane phospholipids, which serve as primary substrates for lipid peroxidation during ferroptosis. Thus, upregulation of the SP1/ACSL4 axis increases cellular susceptibility to ferroptosis by enriching pro-ferroptotic phospholipid substrates. To protect normal ovarian cells, both the ACSL4 inhibitor rosiglitazone (Rosi) and the ferroptosis inhibitor Ferrostatin-1 (Fer-1) effectively mitigate cisplatin-induced ovarian damage and granulosa cell death (Zhang et al., 2024). Conversely, this suggests that chemotherapy-resistant cancer cells may acquire resistance to cisplatin-induced ferroptosis by downregulating the SP1/ACSL4 metabolic pathway, thereby reducing ferroptosis-promoting phospholipid substrates. Furthermore, ferroptosis-suppressive pathways independent of GPX4—such as FSP1-CoQ_10_ and GCH1-BH_4_—also contribute to the defense network of tumor cells (Li et al., 2021). These pathways counteract lipid peroxidation through distinct redox metabolic routes (e.g., reducing CoQ_10_ to antioxidative ubiquinol using NAD(P)H, or directly scavenging free radicals *via* BH_4_), collectively reducing sensitivity to ferroptosis triggered by cisplatin and other agents. In summary, targeting key ferroptosis regulatory nodes such as GPX4, ACSL4, or FSP1 offers dual translational potential: on one hand, it may advance the application of ferroptosis inhibitors in protecting normal cells during cancer therapy; on the other hand, it could provide novel strategies for reversing cisplatin resistance in ovarian cancer by reactivating the ferroptosis process.

### 4.3 Application potential of ferroptosis in the prevention and treatment of ovarian cancer

Beyond established ferroptosis inducers such as GPX4 inhibitors (e.g., RSL3) and system Xc^−^ inhibitors (e.g., erastin), recent studies have identified natural products with ferroptosis-inducing activity. Daphnetin, for instance, has been shown to directly trigger ferroptosis in ovarian cancer cells by inhibiting NQO1 (Ma N. et al., 2024). Its mechanisms include promoting intracellular Fe^2+^ accumulation, elevating ROS levels, inducing lipid peroxidation, and downregulating key proteins such as SLC7A11 and GPX4.Notably, NQO1 is highly expressed in chemotherapy-resistant ovarian cancer tissues, and its overexpression can reverse the ferroptotic effects induced by daphnetin. This suggests that NQO1 is not only relevant for assessing ferroptosis sensitivity but may also serve as a target for overcoming platinum-based drug resistance. Similarly, a novel GPX4 inhibitor, Compound AI-3p, selectively kills cisplatin-resistant ovarian cancer cells by inhibiting the GPX4/GSH axis and inducing ferroptosis (Wang et al., 2026). Combining ferroptosis inducers like daphnetin with cisplatin has been demonstrated to significantly enhance antitumor efficacy both *in vitro* and *in vivo*, while also mitigating systemic toxicity associated with chemotherapy. However, clinical application of these agents has not yet been established.

Ferroptosis-targeted therapy in ovarian cancer still faces multiple challenges and opportunities in clinical translation. In addition to developing more potent and less toxic classic ferroptosis inducers, efforts should expand to include inhibitors targeting non-canonical pathways such as NQO1 and FSP1, as well as their combination with PARP inhibitors, immune checkpoint blockers, and other agents. Further research is needed to elucidate the crosstalk between different modes of cell death within the tumor microenvironment, particularly the regulatory networks connecting ferroptosis, apoptosis, and autophagy, especially at key nodes such as p53.While advancing therapeutic strategies, attention must also be paid to ferroptosis in normal ovarian tissues triggered by chemotherapeutic agents such as cisplatin, which can induce a mitochondrial ROS burst and downregulate GPX4. Exploring the adjunctive value of antioxidants like N-acetylcysteine (NAC) in preserving ovarian function warrants focused investigation.

Besides the above challenges, several additional limitations of ferroptosis-based therapy merit attention. First, ferroptosis sensitivity in ovarian cancer cells is highly context-dependent and is influenced by the genetic background, including TP53 mutations (Han et al., 2024), KRAS mutations, or BAP1 mutations (Zhang Y. et al., 2018), as well as the cellular metabolic state, such as lipid profile and iron storage (Zhou Q. et al., 2024). Second, adaptive resistance: prolonged exposure to low-dose ferroptosis inducers may upregulate NRF2-driven antioxidant genes, including GPX4, FSP1, and GCH1, thereby leading to acquired tolerance, as shown in OSCC models (Han et al., 2022) (Li et al., 2021). Third, whether ferroptosis inducers cause significant toxicity in normal tissues such as the kidney, liver, and nervous system remains largely unknown and requires systematic evaluation. For ovarian cancer patients, especially those who receive multiple lines of chemotherapy, the potential risk of renal and hepatic ferroptosis cannot be ignored. Finally, a lack of predictive biomarkers, such as ACSL4 expression or lipid peroxidation levels, hinders patient selection and the real-time monitoring of treatment responses (Zhou Q. et al., 2024; Wang et al., 2024).

Moving forward, personalized ferroptosis-based treatment regimens require systematic validation using preclinical models such as organoids and PDX to maximize efficacy while minimizing toxicity. Thus, by precisely targeting the metabolic vulnerabilities of drug-resistant cells, ferroptosis-centered strategies not only open new avenues for overcoming chemotherapy resistance in ovarian cancer but also offer dual benefits: improving patient survival outcomes and preserving fertility potential.

## 5 Crosstalk between copper and iron homeostasis in cisplatin-resistant ovarian cancer

**FIGURE 5:**
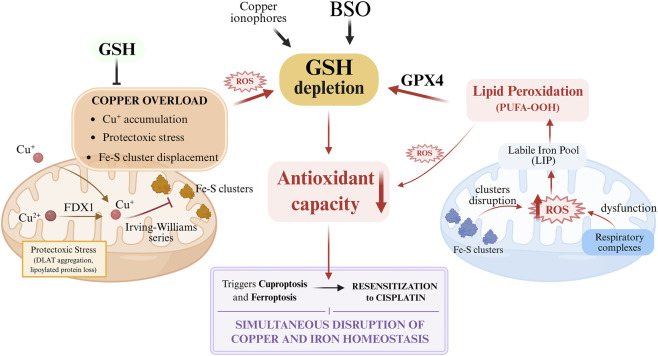
Crosstalk between copper and iron homeostasis in cisplatin-resistant ovarian cancer cells. Copper and iron metabolism are tightly interconnected in cisplatin-resistant ovarian cancer cells, where chronic redox stress and enhanced mitochondrial dependence create a vulnerable state. Glutathione (GSH) serves as a central node in this network by buffering copper-induced toxicity and supporting GPX4-mediated inhibition of lipid peroxidation. Copper ionophores and the GSH synthesis inhibitor BSO promote GSH depletion, thereby facilitating copper overload, mitochondrial Cu^+^ accumulation, proteotoxic stress, and Fe–S cluster displacement through the Irving–Williams effect. Loss of Fe–S cluster integrity leads to respiratory complex dysfunction, increased reactive oxygen species (ROS) production, expansion of the labile iron pool, and activation of iron-dependent lipid peroxidation. In parallel, the system Xc^−^/SLC7A11/GPX4 axis suppresses ferroptosis, whereas FDX1 and copper transporters such as CTR1 and ATP7A/B regulate copper import, distribution, and detoxification. Collectively, simultaneous disruption of copper and iron homeostasis lowers the antioxidant capacity of resistant ovarian cancer cells, triggers cuproptosis and ferroptosis, and may resensitize them to cisplatin (Figure was created with BioRender.com). Abbreviations: BSO, Buthionine sulfoximine; DLAT, Dihydrolipoamide acetyltransferase; FDX1, Ferredoxin 1; GSH, Glutathione; GPX4, Glutathione peroxidase 4; LIP, Labile iron pool; PUFA-OOH, Polyunsaturated fatty acid hydroperoxides; ROS, Reactive oxygen species.

Although cuproptosis and ferroptosis are triggered by distinct metals, they are functionally interconnected through shared redox couples and mitochondrial ligands (Figure 5) (Liu and Chen, 2024; Terzi and Possemato, 2024). This crosstalk may represent a previously underappreciated vulnerability in ovarian cancer cells whose redox balance is already strained by chronic cisplatin stress. GSH sits at the apex of this interplay. As the dominant intracellular thiol, GSH simultaneously acts as a copper chelator that restrains cuproptosis and as the obligatory cofactor for GPX4 that prevents ferroptosis (Liu and Chen, 2024; Terzi and Possemato, 2024). The GSH synthesis inhibitor buthionine sulfoximine (BSO) can therefore relieve the inhibition of both death modes concurrently (Liu and Chen, 2024). Copper ionophores exploit this dual dependency: by delivering copper to mitochondria and depleting GSH, they create a redox environment permissive for both copper-driven proteotoxic stress and iron-catalyzed lipid peroxidation (Liu and Chen, 2024; Li et al., 2023). In colorectal cancer cells, copper overload has been shown to degrade ATP7A and destabilize SLC7A11, thereby coupling copper accumulation to iron-dependent ferroptosis (Li et al., 2023). Whether this mechanism operates in ovarian cancer remains to be investigated.

Mitochondria are the platform where copper and iron metabolism most directly intersect. When copper is in excess, Cu^+^ can displace iron from Fe–S clusters in respiratory complexes *via* the Irving–Williams effect, thereby destroying their catalytic function (Terzi and Possemato, 2024). The displaced iron expands the labile iron pool, fueling Fenton chemistry, whereas iron regulatory proteins sense the Fe–S deficit as iron starvation and paradoxically upregulate TFR1 and ferritinophagy (Terzi and Possemato, 2024). In ovarian cancer cells, which rely heavily on mitochondrial OXPHOS—particularly cisplatin-resistant subpopulations enriched by drug selection—this Fe–S displacement cascade may constitute a metabolic trap. Consistently, the copper ionophore elesclomol exhibits preferential cytotoxicity against ovarian cancer stem-like cells (Wang Q. et al., 2021). The two death pathways can also be activated cooperatively. In primary liver cancer, erastin and sorafenib not only block system Xc^−^–GSH–GPX4 signaling to induce ferroptosis but also prevent FDX1 degradation, thereby promoting cuproptosis (Liu and Chen, 2024). Whether this bidirectional cooperativity operates in ovarian cancer remains to be directly tested; however, the frequent elevation of SLC7A11 and GPX4 in platinum-resistant ovarian cancer suggests that simultaneously targeting both pathways could lower the cell death threshold (Wang Q. et al., 2021). Natural compounds that perturb both copper and iron homeostasis further illustrate this crosstalk. Curcumin triggers features of both ferroptosis and cuproptosis in hepatocellular carcinoma cells (Chauhan et al., 2025), while berberine depletes GSH and inhibits GPX4 in nasopharyngeal carcinoma and may also disturb copper distribution (Chauhan et al., 2025). Whether such dual-modality agents are effective in ovarian cancer requires systematic evaluation.

For ovarian cancer, the clinical relevance of copper–iron crosstalk is underscored by the shared transport machinery governing both cisplatin handling and metal homeostasis. Cisplatin enters cells partly through CTR1 and is sequestered by GSH and ATP7A/B (Li et al., 2023). Thus, the same network that modulates cisplatin sensitivity also controls susceptibility to both cuproptosis and ferroptosis (Terzi and Possemato, 2024). An intervention that disrupts this metal–redox crosstalk could simultaneously activate both death pathways and resensitize ovarian cancer cells to cisplatin. Although direct evidence for copper–iron interplay in ovarian cancer remains limited, its importance across cancer types is increasingly appreciated (Liu and Chen, 2024; Li et al., 2023; Chauhan et al., 2025). We anticipate that further basic and clinical studies will clarify how these two metals jointly influence cisplatin response and open new avenues for therapeutic intervention.

## 6 Conclusion

Although cuproptosis and ferroptosis offer new perspectives for overcoming cisplatin resistance, ovarian cancer‐related evidence and candidate therapeutic strategies are summarized in Tables 2, 3, respectively. However, their translational application still faces several challenges. First, it remains unclear how platinum ions from cisplatin affect the transport and metabolism of copper and iron within ovarian cancer cells. The dynamic changes and potential interactions between copper and iron metabolism across different stages of tumor progression require further exploration.Do these two metals compete with or antagonize each other at the metabolic or redox level? Moreover, clinically relevant methods to assess and stratify the degree of copper and iron dysregulation in tumors are lacking, yet such characterization is essential for understanding the links among metal ion homeostasis, oxidative stress, and cisplatin resistance. Validation of candidate biomarkers—such as serum copper and ceruloplasmin, tumor CTR1/ATP7A expression, FDX1 levels, ferritin, and GPX4 activity—is hampered by a lack of prospective cohorts and standardized detection methods (Table 4). Patient stratification based on copper and iron metabolic signatures (e.g., cuproptosis-related lncRNA models) has not yet been prospectively validated. Second, the heterogeneity of oxidative stress in drug-resistant cells presents another key issue: do subpopulations with distinct metabolic preferences and varying sensitivities to cuproptosis and ferroptosis coexist within the same tumor? How can these subpopulations be identified in vivo—through metabolic imaging or liquid biopsy? Tumor heterogeneity is further reflected in differences between primary and metastatic lesions, as well as across histotypes such as high-grade serous carcinoma *versus* clear cell carcinoma. Third, the targeting and safety profiles of cuproptosis and ferroptosis inducers in cisplatin-resistant ovarian cancer patients need optimization. How can we minimize interference with metal homeostasis and redox balance in normal tissues such as the liver and kidneys? Cuproptosis inducers carry risks of hepatotoxicity, nephrotoxicity, and neurotoxicity, whereas ferroptosis inducers may cause renal tubular injury and pulmonary fibrosis. Finally, a robust biomarker system for clinical translation has yet to be fully established. Are there more sensitive and dynamic metabolic or imaging biomarkers that could be used to predict and monitor treatment response? Beyond biomarker issues, several additional barriers hinder clinical implementation: including unclear regulatory pathways, difficulty in dose optimization for combination therapies, and a lack of industrial support. Moreover, current preclinical models—including common cell lines like A2780 and SKOV3—incompletely represent patient heterogeneity; PDX models retain only partial features of the tumor microenvironment, and organoid models are still at an early stage. Addressing these questions will deepen our understanding of the crosstalk between redox metabolism and regulated cell death networks, and provide a theoretical foundation for designing interventions to reverse cisplatin resistance.

**TABLE 2:** Ovarian-cancer-related studies on cuproptosis/ferroptosis and cisplatin resistance.

Evidence level	Type	Study (first author, year)	Model	Key findings
Preclinical *in vivo*	Cuproptosis	(Zou et al., 2025)	OC cells + mouse xenograft	Cuproptosis inhibits OC proliferation, migration, invasion *via* FDX1; enhances cisplatin sensitivity; cholesterol plays a key role in cytotoxicity
	Ferroptosis	(Zhang et al., 2024)	Rat granulosa cells + SD rats	Fer-1 alleviates cisplatin-induced ferroptosis and ovarian damage by inhibiting iron-dependent lipid peroxidation
Cell-based	Cuproptosis	(Guo et al., 2022)	Cisplatin-resistant OC cells	DSF/Cu overcomes cisplatin resistance at very low doses; highly cytotoxic to resistant cells
	Ferroptosis	(Ma et al., 2024b)	OC cells + mouse xenograft + clinical tissues	Daphnetin induces ferroptosis by inhibiting NQO1; synergizes with cisplatin; NQO1 overexpression confers resistance
	Ferroptosis	(Wang et al., 2026)	Cisplatin-resistant SKOV3/DDP cells and normal IOSE80 cells	Novel GPX4 inhibitor AI-3p selectively kills resistant cells and triggers ferroptosis *via* GPX4/GSH axis
Non-cancerous (Provides mechanistic insight)	Ferroptosis	(Wang et al., 2024)	Ovarian granulosa cells (non-cancerous) + mice	Cisplatin induces ferroptosis *via* SP1/ACSL4 axis; used to model ovarian damage

**TABLE 3:** Candidate therapeutic strategies targeting cuproptosis and ferroptosis in ovarian cancer.

Strategy	Representative agent/Platform	Mechanism	Potential limitations	References
Copper ionophore	Elesclomol and Cu^2+^	Induces cuproptosis *via* FDX1; degrades ATP7A	Potential off-target toxicity	Zou et al. (2025), Gao et al. (2021), Harrington et al. (2020)
	Disulfiram and Cu^2+^	DSF/Cu depletes Fe-S proteins; inhibits efflux pumps	Rapid metabolism *in vivo*	Guo et al. (2023), McMahon et al. (2020), Sauna et al. (2004), Guo et al. (2022)
Cuproptosis nanoplatform	Lipo@CP@DQ NPs	Acid-responsive copper release, GSH depletion, *in situ* chemotherapy	Tumor-specific delivery challenge	Yan et al. (2025)
	GOx@[Cu(tz)]	GSH/glucose depletion, photodynamic therapy	Limited efficacy in hypoxic tumors	Xu et al. (2022)
GPX4 inhibitor	RSL3	Direct GPX4 inhibition	Poor *in vivo* stability	Yang et al. (2014)
System Xc^−^ inhibitor	Erastin	GSH depletion *via* SLC7A11 inhibition	Low solubility	Sato et al. (2018)
Natural product	Daphnetin	NQO1 inhibition; ferroptosis induction	Variable oral bioavailability	Ma et al. (2024b)
Combination	Elesclomol-Cu + cisplatin	Enhances cisplatin uptake; depletes GSH to impair detoxification and sensitize cells to cuproptosis/ferroptosis	Increased systemic toxicity risk; ovarian cancer-specific validation still needed	Zou et al. (2025), Liu and Chen (2024), Li et al. (2023)
	DSF/Cu + cisplatin	Inhibits P-gp; increases cisplatin accumulation; depletes Fe-S proteins	Drug-drug interaction concerns	Guo et al. (2022)
​	Erastin/Sorafenib + Elesclomol-Cu	Ferroptosis inducers inhibit FDX1 degradation and suppress GSH synthesis, thereby enhancing cuproptosis	direct ovarian cancer evidence still lacking	Liu and Chen (2024)

**TABLE 4:** Candidate biomarkers and major translational challenges for cuproptosis- and ferroptosis-based therapies in ovarian cancer.

Category	Candidate biomarker	Challenges	References
Copper status	Serum copper, ceruloplasmin levels	lacking standardized thresholds influenced by diet, inflammation; no established cut-offs for ovarian cancer	Yaman et al. (2007)
Copper transporters	CTR1, ATP7A, ATP7B expression	Intra-tumor heterogeneity; lack of standardized scoring; limited prospective validation	Yoshida et al. (2013), Lukanović et al. (2020), Li et al. (2017), Holzer et al. (2004), Samimi et al. (2004)
Cuproptosis sensitivity	FDX1, LIAS, DLAT expression	No established cut-offs; ovarian-cancer-specific validation lacking	(Tian et al., 2023; Tsvetkov et al., 2022) Zulkifli et al. (2023), Lin et al. (2024), Dreishpoon et al. (2023)
Ferroptosis	GPX4, SLC7A11, ACSL4 expression	Expression varies with genetic background; no established clinical cut-offs	Zhou et al. (2024b), Doll et al. (2017), Yang et al. (2014), Wang et al. (2024), Li et al. (2021), Ma et al. (2024b)
